# The role of income and occupation in the association of education with healthy aging: results from a population-based, prospective cohort study

**DOI:** 10.1186/s12889-015-2504-9

**Published:** 2015-11-25

**Authors:** Christine M. White, Philip D. St. John, Madelon R. Cheverie, Maryam Iraniparast, Suzanne L. Tyas

**Affiliations:** School of Public Health and Health Systems, University of Waterloo, 200 University Avenue West, Waterloo, ON N2L 3G1 Canada; Department of Medicine and Centre on Aging, Section of Geriatric Medicine, University of Manitoba, 820 Sherbrook Street, Winnipeg, MB R3A 1R9 Canada

**Keywords:** Successful aging, Healthy aging, Educational status, Income, Occupations, Gender, Aged, Cohort studies, Logistic models

## Abstract

**Background:**

The beneficial effects of higher education on healthy aging are generally accepted, but the mechanisms are less well understood. Education may influence healthy aging through improved employment opportunities that enhance feelings of personal control and reduce hazardous exposures, or through higher incomes that enable individuals to access better health care or to reside in better neighbourhoods. Income and occupation have not been explored extensively as potential mediators of the effect of education on healthy aging. This study investigates the role of income and occupation in the association between education and healthy aging including potential effect modification by gender.

**Methods:**

Logistic regression was used to explore the association of education, income (perceived income adequacy, life satisfaction with finances) and occupation (occupational prestige) with healthy aging five years later in 946 community-dwelling adults 65+ years from a population-based, prospective cohort study in Manitoba, Canada.

**Results:**

Higher levels of education generally increased the likelihood of healthy aging. After adjusting for education, both income measures, but not occupation, predicted healthy aging among men; furthermore, the association between education and healthy aging was no longer significant. Income and occupation did not explain the significant association between education and healthy aging among women.

**Conclusions:**

Perceived income adequacy and life satisfaction with finances explained the beneficial effects of higher education on healthy aging among men, but not women. Identifying predictors of healthy aging and the mechanisms through which these factors exert their effects can inform strategies to maximize the likelihood of healthy aging.

**Electronic supplementary material:**

The online version of this article (doi:10.1186/s12889-015-2504-9) contains supplementary material, which is available to authorized users.

## Background

Socioeconomic factors are strongly associated with health [1]. Although not universally found [2, 3], the association between education and healthy aging is generally well established, with higher levels of education associated with longer life and delayed disease onset [3–7]. The mechanisms for the association between education and healthy aging, however, remain unclear [8, 9]. Health behaviours and access to health care are frequently implicated in the association between education and healthy aging [8], but do not fully explain the association between education and health [10]. Another way in which education may produce beneficial effects on healthy aging is through improved occupational opportunities and higher income.

Individuals with more education may have better employment opportunities and correspondingly better financial and social status in adulthood [7, 11], which can lead to increased self-esteem, greater life satisfaction, and better health in older adulthood [6]. Education may also affect health by facilitating the choice of and access to a healthier occupation [12], with reduced exposure to hazardous environments and physical strain, or higher levels of personal control and fulfillment [8, 12, 13].

Income has also been associated with health in late life [2]. For example, the proportion of older adults who are classified as ‘high-functioning’ is substantially higher among individuals with high income levels [14]. Similarly, low levels of economic hardship have been associated with good health [8]. Previous research also indicates that the association between education and disability weakens when adjusted for occupation and wealth, supporting a causal model where education influences occupation, which in turn affects wealth [15]. Other studies, however, have not found an association between adequate income and sustained independence [3]. The role of income as a mediator of health rather than simply a predictor of healthy aging has not been extensively explored. Individuals with higher levels of education typically have higher paying jobs, and consequently, greater financial resources; this may increase the likelihood of healthy aging by enabling individuals to access better health care or to reside in better neighbourhoods [6]. Previous research, however, has not consistently found that income contributes to the association between education and healthy aging [11].

Gender is a major determinant of mortality and morbidity, with women known to have longer life expectancies but higher rates of illness and disability [16, 17]. Gender is also associated with educational level [2] and the association between education and health may vary by gender [18, 19]. Thus, there may be gender-specific differences in the effects of education on healthy aging. For example, higher levels of education have been associated with higher levels of physical function among older women, but not among older men; for men, income was a more important predictor of physical function [20]. In contrast, another study reported that women with a college education or higher had more disability than men with no formal education [21]. Gender-specific differences in healthy aging may partially reflect differential socioeconomic experiences between men and women that may be related to education, including labour force participation and financial independence [22]. For example, higher levels of education were related to occupational outcomes in older men but not in older women [23]. Disability rates were also higher for women in the top quintile of wealth, compared to men in the second lowest wealth quintile [21]. While disability was generally least common in those at the highest socioeconomic levels (measured by education, social class and wealth), the association was stronger in men [15].

The aim of this study was to confirm the association between education and healthy aging and to assess income and occupation as potential mediators of this association in a population-based, prospective cohort study of community-dwelling seniors. In order to gain a better understanding of this association in men and women, the study also investigated potential effect modification by gender.

## Methods

### Study population

This study was conducted using data from the Manitoba Study of Health and Aging (MSHA), a prospective cohort study of aging and dementia in community-based and institutionalized adults age 65 years and older [24, 25]. In brief, the MSHA is an expansion of the Canadian Study of Health and Aging [26] in the province of Manitoba, Canada, with similar instruments for data collection and diagnosis but additional measures of social well-being and a sampling frame enriched by rural residents. For the community-based sample, participants were randomly selected from a list provided by the provincial health insurance plan, which provides virtually universal coverage. The sample excluded individuals insured through federal health plans (the military, Royal Canadian Mounted Police, and members of First Nations [North American Indian] residing on reserves), as well as individuals living in a remote, sparsely populated region of the province. Individuals living in correctional institutions, mental health hospitals or personal care homes at baseline were also excluded from the community-based sample. To ensure adequate representation of the oldest individuals, the sample was stratified by Manitoba Health Region and age group (65–74, 75–84, ≥85 years), with deliberate over-sampling of individuals in the two older age strata. Data were collected by personal interview, self-reported questionnaire, and clinical examination in 1991–92; participants were re-contacted in 1996–97.

Of 2,194 individuals who were contacted and eligible at baseline, 1,751 (80 %) agreed to participate. At the five-year follow-up, 573 were ineligible and excluded because of death (n = 371), institutionalization (n = 39), health issues that precluded their participation (n = 86), dementia (n = 43), or inability to be contacted (n = 34). Of the 1,178 eligible participants, 232 were excluded because of refusals (n = 95) or missing data (n = 137), leaving a sample size of 946 (80 %) (refer to Additional file 1). Ethics approval was received from the Faculty of Medicine Committee on the Use of Human Subjects in Research at the University of Manitoba for the MSHA and the Office of Research Ethics at the University of Waterloo for the present study. The study was in adherence with the Declaration of Helsinki.

### Measures

To meet criteria for healthy aging, participants were required to have good function in all of the four components: physical, cognitive, social and psychological health (see Table 1) [27]. The cognitive health component was based on scores from the Modified Mini-Mental State Exam (3MS) [28]. Physical health was assessed using activities of daily living (ADL) and instrumental activities of daily living (IADL) [29, 30]. The social health component used a Terrible-Delightful scale to measure satisfaction with social relationships, and instrumental and emotional support questions to measure availability of social support [31]. The psychological health component included measures of general life satisfaction using the Terrible/Delightful scale [31] and depressive symptoms as indicated by the Centre for Epidemiological Studies Depression (CES-D) scale [32].

**Table 1:** Criteria for healthy aging^a^ by domain

Domain	Criteria for healthy aging
Physical health	• Independent on all 7 ADLs (eating, dressing, taking care of their appearance, walking, getting in/out of bed, bathing, and toileting) and 7 IADLs (shopping, cooking, doing housework, taking medications, handling personal finances, using the telephone, and getting to places out of walking distance) [19, 20]
Cognitive health	• Score of ≥ 78 on the Modified Mini-Mental State Examination (3MS) [18]
Social health	• Positive responses (“mostly satisfied”, “pleased” or “delighted”) on each of 3 questions assessing satisfaction with social relationships (satisfaction with family, friendships, and recreational activities) • Reported availability of instrumental and emotional support (someone they can count on for help and support, someone they can call for help if injured, and someone they can count on to listen when they need to talk) [21]
Psychological health	• Score of <16 on the Center for Epidemiologic Studies Depression (CES-D) scale [22] • “Good” general life satisfaction (i.e., answered “mostly satisfied”, “pleased”, or “delighted” when asked “How do you feel about your life as a whole right now?”) [21]

^a^Criteria must be met in all domains to meet the definition of healthy aging.

ADL, (basic) activities of daily living; IADL, instrumental activities of daily living

Participants were asked to report their highest level of formal education using eleven possible levels (see Table 2). Few participants attained a master’s or doctoral degree and thus these responses were combined. Income was assessed using questions on average monthly household income, perceived income adequacy, and life satisfaction with finances. *Household income* was determined by asking, “What is the average monthly income for your household, including the old age security payment?” *Perceived income adequacy* was evaluated by asking, “How do you think your income and assets currently satisfy your needs?” *Life satisfaction with finances* reflected participants’ satisfaction with their current income and assets according to a terrible/delightful scale. Income categories were combined when warranted by small cell sizes.

**Table 2:** Characteristics of study population at baseline by healthy aging status at follow-up, Manitoba Study of Health and Aging (n = 946)

Characteristics	Healthy aging (n = 359)	Not healthy aging (n = 587)	Total sample (n = 946)
Age, years (mean, SD)*	73.6 (5.3)	76.9 (6.3)	75.7 (6.1)
Gender (% female)	59.6	61.0	60.5
Level of education* (%)			
No formal schooling	0.0	1.7	1.1
Some primary school	7.2	15.3	12.3
Finished primary school	13.1	14.1	13.7
Some secondary or high school	36.5	32.7	34.1
Completed secondary or high school	16.7	17.0	16.9
Some community/technical college	4.7	4.8	4.8
Completed community/technical college	8.9	8.4	8.6
Some university	4.5	2.2	3.0
Bachelor’s degree	5.9	2.7	3.9
Master’s degree or PhD	2.5	1.0	1.6
Monthly household income, $ (mean, SD)*^a^	1921 (1361)	1545 (1154)	1694 (1253)
*Perceived income adequacy (%)*			
Totally inadequate	0.0	0.0	0.0
Not very well/some difficulty	10.0	13.1	12.0
Adequately	63.5	62.0	62.6
Very well	26.5	24.9	25.5
Life satisfaction with finances (%)*			
Not happy	9.5	12.8	11.5
Happy	67.1	72.1	70.2
Very happy	23.4	15.2	18.3
*Occupation (%)*			
Unskilled	15.9	19.9	18.4
Semiskilled	20.3	18.4	19.1
Farmers	19.2	23.7	22.0
Skilled	17.3	13.6	15.0
Technicians and middle management	10.0	10.9	10.6
Professionals	17.3	13.5	14.9

*p < 0.001; ^a^n = 796

Occupational prestige of the participant’s longest job was classified according to Statistics Canada’s Standard Occupational Classifications [33]. Spousal data on longest job was used as a proxy measure for participants who responded that they were a housewife (n = 108), had never worked (n = 19), or could otherwise not be classified (n = 1). Occupation was then grouped into six categories using an established socioeconomic classification system (see Table 2) [34, 35].

### Data analysis

Chi-square and t-tests were used to investigate associations of sample characteristics with healthy aging. Analysis of variance with Scheffé post-hoc tests were used to compare household income with perceived income adequacy and life satisfaction with finances. Logistic regression models were developed to explore the relationship of education at baseline to healthy aging five years later, and to investigate perceived income adequacy, life satisfaction with finances, and occupation as potential mediators of this relationship. All odds ratio (OR) values from these regression models were adjusted for age and gender, and interactions tested. Standard regression diagnostics and multi-collinearity assessments were performed. Analyses were conducted using SAS versions 9.1-9.4 (SAS Institute Inc., Cary, North Carolina).

## Results

### Bivariate analyses

Individuals who met criteria for healthy aging were significantly younger and more educated (see Table 2). They did not differ on perceived income adequacy or occupation, but did differ significantly on life satisfaction with finances. (For characteristics of participants excluded from analyses, refer to Additional file 2. For characteristics of participants at baseline by healthy aging status at baseline, refer to Additional file 3). Overall, 796 of the 946 participants (84.1 %) provided information on household income. Those who met criteria for healthy aging were more likely to have reported household income compared to those who did not meet criteria for healthy aging (87.7 % vs 81.9 %, p <0.05), and more likely to have reported higher levels of this income at baseline (Table 2). Perceived income adequacy and life satisfaction with finances were compared to household income to determine whether they were appropriate proxy measures for income. Both measures were associated with income: mean monthly income rose significantly with each increasing level of both perceived income adequacy and life satisfaction with finances (refer to Additional file 4 for details on mean household income by adequacy and satisfaction level).

### Models with men and women combined

In the multivariate models with men and women combined, education was a significant predictor of healthy aging (Table 3, Model 1). As educational attainment increased, the likelihood of healthy aging also increased (OR = 1.16; 95 % CI = 1.08-1.25). In this model, healthy aging was not significantly associated with gender (OR = 1.03, 95 % CI = 0.78-1.37), but was significantly less likely in older individuals (OR = 0.91, 95 % CI = 0.89-0.93).

**Table 3:** The association of healthy aging at follow-up with baseline education, perceived income adequacy, life satisfaction with finances and occupational prestige among women and men, Manitoba Study of Health and Aging (n = 946)

Healthy aging model^a^	Model 1 OR (95 % CI)	Model 2 OR (95 % CI)	Model 3 OR (95 % CI)	Model 4 OR (95 % CI)	Model 5 OR (95 % CI)	Model 6 OR (95 % CI)	Model 7 OR (95 % CI)	Model 8 OR (95 % CI)	Model 9 OR (95 % CI)
Education^b^	**1.16 (1.08-1.25)**		**1.16 (1.08-1.25)**		**1.14 (1.05-1.23)**		**1.16 (1.06-1.27)**		**1.15 (1.05-1.26)**
*Perceived income adequacy*									
With some difficulty/not very well^c^									
Adequately		1.49 (0.96-2.34)	1.38 (0.89-2.17)					1.13 (0.66-1.95)	1.09 (0.64-1.88)
Very well		1.51 (0.93-2.47)	1.23 (0.75-2.05)					0.86 (0.46-1.59)	0.78 (0.42-1.46)
*Life satisfaction with finances*									
Not happy^c^									
Happy				1.50 (0.96-2.37)	1.43 (0.91-2.26)			1.42 (0.83-2.48)	1.44 (0.84-2.51)
Very happy				**2.61 (1.56-4.44**)	**2.22 (1.31-3.81)**			**2.67 (1.40-5.17)**	**2.58 (1.35-5.01)**
*Occupational prestige*									
Unskilled^c^									
Semiskilled						1.44 (0.92-2.26)	1.37 (0.88-2.16)	1.46 (0.93-2.30)	1.39 (0.88-2.20)
Farmers						1.21 (0.77-1.89)	1.22 (0.78-1.92)	1.24 (0.79-1.95)	1.24 (0.79-1.97)
Skilled						**1.69 (1.05-2.73)**	1.47 (0.91-2.40)	1.61 (0.99-2.61)	1.42 (0.87-2.33)
Technicians and middle management						1.36 (0.79-2.32)	1.15 (0.66-1.99)	1.32 (0.76-2.28)	1.13 (0.65-1.98)
Professionals						**1.93 (1.20-3.13)**	1.18 (0.66-2.08)	**1.79 (1.10-2.92)**	1.15 (0.64-2.04)

CI = confidence interval; OR = odds ratio

**Bold** denotes p < 0.05

^a^Adjusted for age and gender

^b^Level of educational attainment (10 levels)

^c^Reference category

Perceived income adequacy was not significantly associated with healthy aging (Table 3, Model 2). Moreover, the association between education and healthy aging was not weakened when perceived income adequacy was considered (Table 3, Model 3). There was no significant interaction between perceived income adequacy and education in the likelihood of healthy aging (data not shown).

Life satisfaction with finances was significantly associated with healthy aging, but only for those reporting they were ‘very happy’ (OR = 2.61; 95 % CI = 1.56-4.44) (Table 3, Model 4); this significant association remained (OR = 2.22; 95 % CI = 1.31-3.81) when adjusted for education (Table 3, Model 5). The association between education and healthy aging weakened slightly (from OR = 1.16 to OR = 1.14) when life satisfaction with finances was considered.

Occupational prestige was significantly associated with healthy aging for those who were in the “skilled” and “professionals” occupational groups (Table 3, Model 6). Any impact of occupational prestige on healthy aging disappeared, however, once the effect of education was considered (Table 3, Model 7).

When the effects of perceived income adequacy, life satisfaction with finances, and occupation were considered together in the absence of education, only the highest level of life satisfaction with finances (very happy) (OR = 2.67; 95 % CI: 1.40-5.17) and the highest occupational level (professionals) (OR = 1.79; 95 % CI: 1.10-2.92) were significant predictors of healthy aging (Table 3, Model 8). When education was added to this model, however, only the highest level of life satisfaction with finances remained a significant predictor of healthy aging (OR = 2.58; 95 % CI: 1.35-5.01) (Table 3, Model 9). There was no significant interaction between occupation and perceived income adequacy or between occupation and life satisfaction with finances (data not shown).

The results of analyses with household income and imputed household income were very similar to the results described above for perceived income adequacy and life satisfaction with finances. Refer to Additional file 5 for models examining the association of healthy aging with household income for those who provided these data (n = 796), and Additional file 6 for similar models that included imputed values for missing household income (n = 946). Given concerns of bias related to missing household income data as well as concerns with imputing income values, all remaining models focus on perceived income adequacy and life satisfaction with finances as measures of income.

### Models stratified by gender

There was a significant interaction of gender with perceived income adequacy and with life satisfaction with finances, but not with occupational prestige. Logistic regression models were thus also examined for men and women separately, with age as a covariate.

A similar pattern of results to that described above (a significant association between education and healthy aging in all models) was found for women (Table 4). Furthermore, when the effects of perceived income adequacy, life satisfaction with finances, and occupation were considered together with education, only education was a significant predictor of healthy aging for women (Table 4, Model 9).

**Table 4:** The association of healthy aging at follow-up with baseline education, perceived income adequacy, life satisfaction with finances and occupational prestige among women, Manitoba Study of Health and Aging (n = 572)

Healthy aging model^a^	Model 1 OR (95 % CI)	Model 2 OR (95 % CI)	Model 3 OR (95 % CI)	Model 4 OR (95 % CI)	Model 5 OR (95 % CI)	Model 6 OR (95 % CI)	Model 7 OR (95 % CI)	Model 8 OR (95 % CI)	Model 9 OR (95 % CI)
Education^b^	**1.18 (1.07-1.31)**		**1.17 (1.06-1.30)**		**1.16 (1.04-1.28)**		**1.23 (1.08-1.39)**		**1.20 (1.06-1.37)**
*Perceived income adequacy*									
With some difficulty/not very well^c^									
Adequately		**1.77 (1.03-3.14)**	1.63 (0.94-2.90)					1.78 (0.92-3.55)	1.69 (0.87-3.39)
Very well		**2.01 (1.09-3.78)**	1.63 (0.87-3.13)					1.50 (0.70-3.32)	1.33 (0.61-2.98)
*Life satisfaction with finances*									
Not happy^c^									
Happy				1.25 (0.72-2.22)	1.16 (0.66-2.07)			0.89 (0.45-1.79)	0.90 (0.45-1.82)
Very happy				**2.41 (1.25-4.72**)	**2.01 (1.03-4.00)**			1.81 (0.79-4.19)	1.76 (0.76-4.10)
*Occupational prestige*									
Unskilled^c^									
Semiskilled						1.35 (0.79-2.30)	1.29 (0.75-2.23)	1.36 (0.80-2.35)	1.31 (0.76-2.28)
Farmers						1.39 (0.78-2.48)	1.35 (0.75-2.42)	1.51 (0.84-2.73)	1.47 (0.81-2.67)
Skilled						1.54 (0.83-2.88)	1.17 (0.61-2.25)	1.42 (0.75-2.69)	1.12 (0.57-2.17)
Technicians and middle management						1.07 (0.51-2.21)	0.88 (0.41-1.84)	1.04 (0.49-2.16)	0.88 (0.41-1.86)
Professionals						1.67 (0.93-3.04)	0.89 (0.43-1.81)	1.52 (0.83-2.81)	0.88 (0.42-1.81)

CI = confidence interval; OR = odds ratio **Bold** denotes p < 0.05

^a^Adjusted for age

^b^Level of educational attainment (10 levels)

^c^Reference category

In contrast, for men the association between education and healthy aging became non-significant when occupation was considered (OR = 1.09; 95 % CI: 0.95-1.25) (Table 5, Model 7). The impact of education remained non-significant when the effects of both income measures (perceived income adequacy, life satisfaction with finances) and occupation were considered together (OR = 1.09; 95 % CI: 0.95-1.26); however, perceived income adequacy and life satisfaction with finances both became significant predictors of healthy aging in this model (Table 5, Model 9).

**Table 5:** The association of healthy aging at follow-up with baseline education, perceived income adequacy, life satisfaction with finances and occupational prestige among men, Manitoba Study of Health and Aging (n = 374)

Healthy Aging Model^a^	Model 1 OR (95 % CI)	Model 2 OR (95 % CI)	Model 3 OR (95 % CI)	Model 4 OR (95 % CI)	Model 5 OR (95 % CI)	Model 6 OR (95 % CI)	Model 7 OR (95 % CI)	Model 8 OR (95 % CI)	Model 9 OR (95 % CI)
Education^b^	**1.14 (1.03-1.28)**		**1.16 (1.04-1.30)**		**1.12 (1.01-1.26)**		1.09 (0.95-1.25)		1.09 (0.95-1.26)
*Perceived income adequacy*									
With some difficulty/not very well^c^									
Adequately		1.02 (0.48-2.21)	0.96 (0.45-2.07)					0.39 (0.13-1.04)	0.37 (0.13-1.01)
Very well		0.90 (0.40-2.05)	0.74 (0.32-1.71)					**0.24 (0.08-0.72)**	**0.22 (0.07-0.68)**
*Life satisfaction with finances*									
Not happy^c^									
Happy				2.02 (0.97-4.48)	1.97 (0.95-4.37)			**3.88 (1.46-11.64)**	**3.95 (1.49-11.89)**
Very happy				**3.04 (1.30-7.47**)	**2.60 (1.10-6.46)**			**6.79 (2.20-23.16)**	**6.66 (2.16-22.76)**
*Occupational prestige*									
Unskilled^c^									
Semiskilled						1.70 (0.75-3.94)	1.62 (0.71-3.76)	1.64 (0.70-3.84)	1.54 (0.66-3.63)
Farmers						1.09 (0.53-2.28)	1.11 (0.54-2.35)	0.98 (0.46-2.10)	1.00 (0.47-2.14)
Skilled						1.99 (0.93-4.34)	1.92 (0.90-4.21)	1.98 (0.91-4.40)	1.91 (0.87-4.25)
Technicians and middle management						1.77 (0.77-4.11)	1.60 (0.68-3.77)	1.70 (0.73-4.03)	1.52 (0.63-3.67)
Professionals						**2.60 (1.14-6.05)**	1.93 (0.74-5.14)	**2.41 (1.04-5.74)**	1.77 (0.66-4.81)

CI = confidence interval; OR = odds ratio **Bold** denotes p < 0.05

^a^Adjusted for age

^b^Level of educational attainment (10 levels)

^c^Reference category

## Discussion

Consistent with previous literature [6, 8, 11], education was a significant predictor of healthy aging in models that combined men and women. As educational attainment increased, so did the likelihood of healthy aging. The goal of this study was to examine the role of income and occupation as possible mediators of the relationship between educational attainment and healthy aging. In models that combined men and women, perceived income adequacy was not significantly related to healthy aging. Among men, however, there was a negative gradient between perceived income adequacy and healthy aging when the effects of income and occupation were considered together in the presence of education: men who thought their income met their needs “very well” were, unexpectedly, less likely to meet criteria for healthy aging compared to those who were at least somewhat dissatisfied with how their income met their needs. Unlike the perceived income adequacy measure, there was a significant positive gradient between life satisfaction with finances and healthy aging in models of men and women separately as well as combined (Tables 3, 4 and 5, Models 4 and 5).

The difference in significant predictors for models with or without education indicates that while education explains the influence of occupation on healthy aging for men, it does not fully explain the influence of perceived income adequacy and life satisfaction with finances on healthy aging, leaving the possibility open that these variables could act as mediators. Additionally, the findings suggest that among men, perceived income adequacy and life satisfaction with finances are more important predictors of healthy aging than education. For women, however, education remains the more influential predictor of healthy aging.

Overall, the study provides evidence that income may mediate the relationship between education and healthy aging among men, but not women, consistent with previous research indicating that, among men, wealth was a more important predictor of physical function than education [20]. There was little evidence, however, that occupation mediates this relationship for either men or women. This conclusion was drawn based on criteria [36] of eliminating or at least reducing the association between education and healthy aging. Together, perceived income adequacy and life satisfaction with finances eliminated the significant association between education and healthy aging among men. In addition, the effects of occupation on healthy aging disappeared for men after education was considered, providing support for a causal model where education influences occupation, which in turn influences financial resources [15]. In contrast, for women, our findings of a strong and persistent influence of education on healthy aging supports the pre-eminence of education over income and occupation as predictors of healthy aging for women.

The differences in our findings by gender may relate to differences in labour force participation and domestic responsibilities between men and women [37], especially among this older cohort, and may not hold for younger cohorts. A substantial proportion of women in this study were homemakers, and men may have been more likely to provide the primary source of income for their families. Subsequently, the men may have placed more importance on finances and their health may have been more affected by financial stress than women. Indeed, previous research has shown that men have significantly higher levels of financial stress than women [22].

Based on the negative gradient observed between perceived income adequacy and healthy aging, one might speculate that men who believe their income meets their needs may have had higher-paying jobs that negatively affected their health. Alternatively, one could argue that individuals who are healthier may have greater financial needs (e.g., travel) than those less healthy in a sample who all had health insurance coverage; those who are less healthy may, in comparison, be more easily satisfied with how their finances meet their needs.

The difference in the direction of the gradient observed for perceived income adequacy and life satisfaction with finances was an unexpected finding, and highlights that these measures have some important conceptual differences. Although these measures are both based on subjective income assessments, perceived income adequacy reflects how their income meets their needs, while life satisfaction with finances measures more general satisfaction with finances. Future research could examine the reasons for these differences, such as psychological and lifestyle-related factors.

Perceived income adequacy and life satisfaction with finances were used as proxy measures of income because a substantial proportion of participants (15.9 %) did not provide information on household income. This level of non-response is similar to that found in other studies [38]. In addition, response rates may differ by income level, indicating the potential for further bias [38]. Use of the household income variable in our study would have created a biased analytic sample as individuals who provided household income differed significantly by healthy aging status. Those who met criteria for healthy aging were more likely to have provided household income information than those who did not meet criteria for healthy aging. Thus, use of alternate measures of income (perceived income adequacy and life satisfaction with finances) was necessary. Although perceived income adequacy and life satisfaction with finances are conceptually different from income, these subjective income measures may be important in providing a perspective independent of income in dollars. This is supported by our finding of a significant association of healthy aging with life satisfaction with finances and perceived income adequacy for men. In addition, sensitivity analyses adding household income to the full model produced the same pattern of results as the analysis without income (Table 3, Model 9), and income was not a statistically significant predictor once education was included in the model (refer to Additional file 5, Model 3 and Additional file 6, Model 3). Our results also indicate that the measure of income that is used can influence the observed association with healthy aging.

Whereas education and income reflect resources, occupational prestige is a direct measure of social standing. It has been associated with measures such as self-reported health, with higher prestige associated with reduced likelihood of poor or fair self-reported health [39]. Limitations related to the occupational prestige measure, however, should be noted. In this older cohort, women were less likely to work outside the home and employment may have been more stable. These changes may decrease the generalizability of our findings to future older adults. The occupation classification system was last updated in the 1980s while the data were collected in the 1990s. Although the levels of prestige associated with certain occupations may have changed over that decade, most participants were already retired and thus earlier classification systems may be most relevant.

Another potential limitation is that healthy aging was defined as a dichotomous variable (presence vs. absence). Although commonly done, alternate definitions of healthy aging using multiple levels or as a continuous index in future studies may provide a more detailed picture of this construct and its predictors.

The results may not be fully generalizable to all populations. This cohort of older adults spent their childhood and early adulthood during the Depression and the Second World War. The education they received and their early careers may have been shaped by those experiences. These formative experiences may have been quite different for other groups of people in other places and other times. Similarly, the gender differences we observed may have been unique to this group of people.

A substantial portion of the baseline sample was deceased, institutionalized or otherwise not eligible to participate at follow-up. Attrition through death is inevitable in longitudinal research, particularly in the aging field, but is important to note as it has the potential to produce bias given that mortality and institutionalization do not occur randomly. For example, those who were dead or institutionalized had lower levels of education than others in the sample (see Additional file 2). These systematic differences may lead to problems generalizing the findings to a broader population of older adults, but they do reflect the selective sample of older adults able to remain the community.

This study was based on data from a population-based, prospective cohort study with a high response rate. Additional study strengths included using two different proxy measures of income (life satisfaction with finances and perceived income adequacy) as well as comparisons of these proxy measures with household income and sensitivity analyses. The study used a multidimensional definition of healthy aging, incorporating physical, cognitive, social, and psychological domains of health. It is generally recognized that absence of disease and good mental health are critical aspects of successful aging, but that overall life satisfaction, social participation, personal growth, and factors identified as important by the general population should also be considered [40–42]. This study utilized a definition of healthy aging that comprised a wide breadth of measures and thus captured healthy aging in a manner consistent with this broader definition.

Future research directions include examining other potential mechanisms by which education may be associated with healthy aging, especially in women. Such mediating factors could include greater engagement in productive activity, perceived control, social support, health beliefs, health service use and cognitive reserve. This area of inquiry is complex. Associations between childhood opportunities, educational attainment, occupation, and income may all be important and inter-related. Furthermore, they may operate at different stages of life. Finally, different cohorts may experience these effects differently. Access to education, job security, and income can change considerably over time, as can their associations with gender. Further study should consider a life course approach following large populations over long time frames.

## Conclusions

Predictors of healthy aging such as education are often set early in life, and thus have important implications for policy interventions. The overall link between education and healthy aging observed in this and other studies supports increased, or at least maintained, expenditures for education. The mechanism by which education exerts its effects on healthy aging among women is not yet clear. Income and occupation do not appear to mediate this relationship. However, among men, perceived income adequacy and life satisfaction with finances appear to mediate the association between education and healthy aging. Further exploration is needed to identify mechanisms through which education influences healthy aging in women. Identifying predictors of healthy aging and the mechanisms through which these factors exert their effects could help guide strategies to maximize the likelihood of healthy aging for all.

## Additional files

Additional file 1:
**Derivation of analytic sample, Manitoba Study of Health and Aging.** (DOCX 44 kb) Additional file 2:
**Characteristics of study population at baseline by healthy aging status at follow-up, Manitoba Study of Health and Aging (n=1,751).** (DOCX 38 kb) Additional file 3:
**Characteristics of study population at baseline by healthy aging status at baseline, Manitoba Study of Health and Aging (n=896).** (DOCX 19 kb) Additional file 4:
**Mean monthly household income by levels of perceived income adequacy and life satisfaction with finances at baseline, Manitoba Study of Health and Aging (n=796).** (DOCX 35 kb) Additional file 5:
**The association of healthy aging at follow-up with baseline education, household Income, perceived income adequacy, life satisfaction with finances and occupational prestige, Manitoba Study of Health and Aging (n=796).** (DOCX 36 kb) Additional file 6:
**The association of healthy aging at follow-up with baseline education, household income, perceived Income adequacy, life satisfaction with finances and occupational prestige, Manitoba Study of Health and Aging (n=946).** (DOCX 36 kb)
