# Supplementary material for: The role of income and occupation in the association of education with healthy aging: results from a population-based, prospective cohort study
Source: BMC Public Health. 2015 Nov 25;15:1181. doi: 10.1186/s12889-015-2504-9 (PMC4660771; doi:10.1186/s12889-015-2504-9)
Supplement: Additional file 1: — Derivation of analytic sample, Manitoba Study of Health and Aging. (DOCX 44 kb) [file 12889_2015_2504_MOESM1_ESM.docx]

**ADDITIONAL FILE 1**

**Derivation of analytic sample, Manitoba Study of Health and Aging**

Baseline 1991/92 Eligible and Contacted for Screening

**1,751**

Follow-up 1996/97 Deceased – 371

Institutionalized – 39

Hospitalized/too sick/
hearing or speech problems – 86

Dementia – 43

No contact – 34

Eligible Sample at Time 2

**1,178**

Refusals – 95

Missing data – 137

Analytic Sample at Time 2

**946**
