# Supplementary material for: The role of income and occupation in the association of education with healthy aging: results from a population-based, prospective cohort study
Source: BMC Public Health. 2015 Nov 25;15:1181. doi: 10.1186/s12889-015-2504-9 (PMC4660771; doi:10.1186/s12889-015-2504-9)
Supplement: Additional file 2: — Characteristics of study population at baseline by healthy aging status at follow-up, Manitoba Study of Health and Aging (n=1,751). (DOCX 38 kb) [file 12889_2015_2504_MOESM2_ESM.docx]

**ADDITIONAL FILE 2**

**Characteristics of Study Population at Baseline by Healthy Aging Status at Follow-up, Manitoba Study of Health and Aging (n=1,751)**

| **Characteristics** | **Healthy  Aging  (n=359)** | **Not Healthy Aging (n=587)** | **Institutionalized (n=39)** | **Dead  (n=371)** | **Missing (n=395)** |
| --- | --- | --- | --- | --- | --- |
| Age, years (mean, SD)*** | 73.6 (5.3) | 76.9 (6.3) | 83.8 (6.3) | 80.9 (7.2) | 78.2 (7.7)^1^ |
| Gender (% female)*** | 59.6 | 61.0 | 66.7 | 48.5 | 62.5 |
| *Level of Education**** |  |  |  |  |  |
| No formal schooling | 0.0 | 1.7 | 0.0 | 2.8 | 3.8 |
| Some primary school | 7.2 | 15.3 | 29.0 | 19.4 | 20.6 |
| Finished primary school | 13.1 | 14.1 | 5.3 | 18.3 | 12.9 |
| Some secondary or high school | 36.5 | 32.7 | 42.1 | 31.1 | 33.2 |
| Completed secondary or high school | 16.7 | 17.0 | 10.5 | 10.6 | 12.9 |
| Some community or technical college | 4.7 | 4.8 | 2.6 | 3.3 | 4.3 |
| Completed community or technical college | 8.9 | 8.4 | 5.3 | 6.9 | 5.4 |
| Some university | 4.5 | 2.2 | 2.6 | 4.4 | 3.2 |
| Bachelor’s degree | 5.9 | 2.7 | 2.6 | 2.2 | 3.0 |
| Master’s degree or PhD | 2.5 | 1.0 | 0.0 | 0.8 | 0.8 |
| Monthly Household Income^2^, $  (mean, SD)*** | 1921  (1360) | 1545  (1154) | 1119  (471) | 1351  (1032) | 1460  (1225) |
| *Perceived Income Adequacy (%)** |  |  |  |  |  |
| Not very well/some difficulty | 10.0 | 13.1 | 5.1 | 14.7 | 14.8 |
| Adequately | 63.5 | 62.0 | 53.9 | 55.4 | 64.5 |
| Very well | 26.5 | 24.9 | 41.0 | 29.9 | 20.7 |
| *Life Satisfaction with Finances (%)*** |  |  |  |  |  |
| Not happy | 9.5 | 12.8 | 12.8 | 12.5 | 17.3 |
| Happy | 67.1 | 72.1 | 71.8 | 71.8 | 64.8 |
| Very happy | 23.4 | 15.2 | 15.4 | 15.8 | 17.9 |
| *Occupation (%)* |  |  |  |  |  |
| Professionals | 17.3 | 13.5 | 13.2 | 15.2 | 12.0 |
| Technicians and Middle Management | 10.0 | 10.9 | 10.5 | 12.0 | 7.9 |
| Skilled | 17.3 | 13.6 | 15.8 | 14.4 | 16.8 |
| Farmers | 19.2 | 23.7 | 23.7 | 22.3 | 20.4 |
| Semiskilled | 20.3 | 18.4 | 21.0 | 17.9 | 24.4 |
| Unskilled | 15.9 | 19.9 | 15.8 | 18.2 | 18.6 |

*p<0.05; **p<.01; ***p<.001

^1^n=394

^2^n=1378 (healthy aging n=315; not healthy aging n=481; institutionalized n=31; dead n=285; missing n=266)
