# Supplementary material for: The role of income and occupation in the association of education with healthy aging: results from a population-based, prospective cohort study
Source: BMC Public Health. 2015 Nov 25;15:1181. doi: 10.1186/s12889-015-2504-9 (PMC4660771; doi:10.1186/s12889-015-2504-9)
Supplement: Additional file 3: — Characteristics of study population at baseline by healthy aging status at baseline, Manitoba Study of Health and Aging (n=896). (DOCX 19 kb) [file 12889_2015_2504_MOESM3_ESM.docx]

**ADDITIONAL FILE 3**

**Characteristics of Study Population at Baseline by Healthy Aging Status at Baseline, Manitoba Study of Health and Aging (n=896)**

| **Characteristics** | **Healthy  Aging  (n=398)** | **Not Healthy Aging (n=498)** | **Total  Sample (n=896)** |
| --- | --- | --- | --- |
| Age, years (mean, SD)* | 74.6 (6.0) | 76.4 (6.2) | 75.6 (6.2) |
| Gender (% female) | 57.5 | 62.7 | 60.4 |
| *Level of Education (%)** |  |  |  |
| No formal schooling | 0.0 | 2.0 | 1.1 |
| Some primary school | 9.3 | 15.3 | 12.6 |
| Finished primary school | 12.1 | 15.9 | 14.2 |
| Some secondary or high school | 36.2 | 32.1 | 33.9 |
| Completed secondary or high school | 15.8 | 16.7 | 16.3 |
| Some community/technical college | 4.5 | 5.0 | 4.8 |
| Completed community/technical college | 10.8 | 7.0 | 8.7 |
| Some university | 4.3 | 2.4 | 3.2 |
| Bachelor’s degree | 4.5 | 2.8 | 3.6 |
| Master’s degree or PhD | 2.5 | 0.8 | 1.6 |
| Monthly Household Income, $  (mean, SD)*^1^ | 1960 (1494) | 1499 (1013) | 1705 (1270) |
| *Perceived Income Adequacy (%)** |  |  |  |
| Totally inadequate | 0.0 | 0.0 | 0.0 |
| Not very well/some difficulty | 5.8 | 16.5 | 11.7 |
| Adequately | 64.8 | 60.8 | 62.6 |
| Very well | 29.4 | 22.7 | 25.7 |
| *Life Satisfaction with Finances (%)** |  |  |  |
| Not happy | 6.3 | 15.1 | 11.2 |
| Happy | 70.4 | 70.5 | 70.4 |
| Very happy | 23.4 | 14.5 | 18.4 |
| *Occupation (%)* |  |  |  |
| Unskilled | 16.3 | 19.7 | 18.2 |
| Semiskilled | 17.8 | 20.1 | 19.1 |
| Farmers | 21.1 | 22.7 | 22.0 |
| Skilled | 16.3 | 14.7 | 15.4 |
| Technicians and Middle Management | 12.1 | 9.4 | 10.6 |
| Professionals | 16.3 | 13.5 | 14.7 |

*p<0.001 at baseline and follow-up, unless otherwise noted.

^1^ n=757
