# Supplementary material for: The role of income and occupation in the association of education with healthy aging: results from a population-based, prospective cohort study
Source: BMC Public Health. 2015 Nov 25;15:1181. doi: 10.1186/s12889-015-2504-9 (PMC4660771; doi:10.1186/s12889-015-2504-9)
Supplement: Additional file 4: — Mean monthly household income by levels of perceived income adequacy and life satisfaction with finances at baseline, Manitoba Study of Health and Aging (n=796). (DOCX 35 kb) [file 12889_2015_2504_MOESM4_ESM.docx]

**ADDITIONAL FILE 4**

**Mean Monthly Household Income by Levels of Perceived Income Adequacy and Life Satisfaction with Finances at Baseline, Manitoba Study of Health and Aging (n=796)**

| **Measure** | **Mean Monthly Household Income ($)** | **Standard  Deviation ($)** |
| --- | --- | --- |
| *Perceived Income Adequacy* |  |  |
| Not very well / some difficulty | 1100.92 ^1a^ | 546.31 |
| Adequately | 1525.61 ^b^ | 998.42 |
| Very well | 2346.93 ^c^ | 1675.41 |
| *Life Satisfaction with Finances* |  |  |
| Not happy | 1219.33 ^a^ | 644.53 |
| Happy | 1594.91 ^b^ | 1155.31 |
| Very happy | 2349.25 ^c^ | 1597.66 |

^1^ Different letters indicate statistically significant differences between levels at p<0.05.
