# Supplementary material for: The role of income and occupation in the association of education with healthy aging: results from a population-based, prospective cohort study
Source: BMC Public Health. 2015 Nov 25;15:1181. doi: 10.1186/s12889-015-2504-9 (PMC4660771; doi:10.1186/s12889-015-2504-9)
Supplement: Additional file 5: — The association of healthy aging at follow-up with baseline education, household Income, perceived income adequacy, life satisfaction with finances and occupational prestige, Manitoba Study of Health and Aging (n=796). (DOCX 36 kb) [file 12889_2015_2504_MOESM5_ESM.docx]

**ADDITIONAL FILE 5**

**The Association of Healthy Aging at Follow-up with Baseline Education, Household Income, Perceived Income Adequacy, Life Satisfaction with Finances and Occupational Prestige, Manitoba Study of Health and Aging (n=796)**

| **Healthy Aging Model^1^** | **Model 1** OR  (95% CI) | **Model 2** OR  (95% CI) | **Model 3** OR  (95% CI) |
| --- | --- | --- | --- |
| Education^2^ |  | **1.15 (1.06-1.25)** | **1.16 (1.05-1.29)** |
| Household Income^3^ | **1.02 (1.01-1.03)** | 1.01 (0.99-1.02) | 1.01 (0.99-1.02) |
| *Perceived Income Adequacy* |  |  |  |
| With some difficulty/   not very well^4^ |  |  |  |
| Adequately |  |  | 1.05 (0.59-1.91) |
| Very well |  |  | 0.70 (0.35-1.39) |
| *Life Satisfaction with  Finances* |  |  |  |
| Not happy^4^ |  |  |  |
| Happy |  |  | 1.54 (0.85-2.85) |
| Very Happy |  |  | **2.89 (1.42-6.00)** |
| *Occupational Prestige* |  |  |  |
| Unskilled^4^ |  |  |  |
| Semiskilled |  |  | 1.61 (0.99-2.65) |
| Farmers |  |  | 1.32 (0.81-2.17) |
| Skilled |  |  | 1.30 (0.76-2.24) |
| Technicians and  Middle Management |  |  | 1.29 (0.70-2.40) |
| Professionals |  |  | 1.07 (0.56-2.02) |

CI=confidence interval; OR=odds ratio

**Bold** denotes p<0.05

^1^ Adjusted for age and gender

^2^ Level of educational attainment (10 levels)

^3^ Per $100

^4^ Reference category
