# Supplementary material for: The role of income and occupation in the association of education with healthy aging: results from a population-based, prospective cohort study
Source: BMC Public Health. 2015 Nov 25;15:1181. doi: 10.1186/s12889-015-2504-9 (PMC4660771; doi:10.1186/s12889-015-2504-9)
Supplement: Additional file 6: — The association of healthy aging at follow-up with baseline education, household income, perceived Income adequacy, life satisfaction with finances and occupational prestige, Manitoba Study of Health and Aging (n=946). (DOCX 36 kb) [file 12889_2015_2504_MOESM6_ESM.docx]

**ADDITIONAL FILE 6**

**The Association of Healthy Aging at Follow-up with Baseline Education, Household Income, Perceived Income Adequacy, Life Satisfaction with Finances and Occupational Prestige, Manitoba Study of Health and Aging (n=946)**

| **Healthy Aging Model^1^** | **Model 1** OR  (95% CI) | **Model 2** OR  (95% CI) | **Model 3** OR  (95% CI) |
| --- | --- | --- | --- |
| Education^2^ |  | **1.14 (1.06-1.23)** | **1.14 (1.04-1.25)** |
| Household Income^3^ | **1.02 (1.01-1.03)** | 1.01 (0.99-1.03) | 1.01 (0.99-1.03) |
| *Perceived Income Adequacy* |  |  |  |
| With some difficulty/   not very well^4^ |  |  |  |
| Adequately |  |  | 1.06 (0.62-1.83) |
| Very well |  |  | 0.72 (0.38-1.36) |
| *Life Satisfaction with  Finances* |  |  |  |
| Not happy^4^ |  |  |  |
| Happy |  |  | 1.43 (0.83-2.49) |
| Very Happy |  |  | **2.50 (1.30-4.86)** |
| *Occupational Prestige* |  |  |  |
| Unskilled^4^ |  |  |  |
| Semiskilled |  |  | 1.39 (0.88-2.20) |
| Farmers |  |  | 1.25 (0.80-1.98) |
| Skilled |  |  | 1.40 (0.85-2.29) |
| Technicians and  Middle Management |  |  | 1.08 (0.62-1.90) |
| Professionals |  |  | 1.10 (0.62-1.97) |

CI=confidence interval; OR=odds ratio

**Bold** denotes p<0.05

^1^ Adjusted for age and gender

^2^ Level of educational attainment (10 levels)

^3^ Per $100; with imputed income values for 150 cases with non-reported income

^4^ Reference category
